# Effectively processing medical term queries on the UMLS Metathesaurus by layered dynamic programming

**DOI:** 10.1186/1755-8794-7-S1-S11

**Published:** 2014-05-08

**Authors:** Kaiyu Ren, Albert M Lai, Aveek Mukhopadhyay, Raghu Machiraju, Kun Huang, Yang Xiang

**Affiliations:** 1 Department of Biomedical Informatics, the Ohio State University, Columbus, OH 43210, USA; 2 Department of Computer Science and Engineer, the Ohio State University, Columbus, OH 43210, USA

## Abstract

**Background:**

Mapping medical terms to standardized UMLS concepts is a basic step for leveraging biomedical texts in data management and analysis. However, available methods and tools have major limitations in handling queries over the UMLS Metathesaurus that contain inaccurate query terms, which frequently appear in real world applications.

**Methods:**

To provide a practical solution for this task, we propose a layered dynamic programming mapping (LDPMap) approach, which can efficiently handle these queries. LDPMap uses indexing and two layers of dynamic programming techniques to efficiently map a biomedical term to a UMLS concept.

**Results:**

Our empirical study shows that LDPMap achieves much faster query speeds than LCS. In comparison to the UMLS Metathesaurus Browser and MetaMap, LDPMap is much more effective in querying the UMLS Metathesaurus for inaccurately spelled medical terms, long medical terms, and medical terms with special characters.

**Conclusions:**

These results demonstrate that LDPMap is an efficient and effective method for mapping medical terms to the UMLS Metathesaurus.

## Background

Efficiently processing and managing biomedical text data is one of the major tasks in many medical informatics applications. Biomedical text analysis tools, such as MetaMap [1] and cTAKES [2], have been developed to extract and analyze medical terms from biomedical text. However, medical terms often have multiple names, which make the analysis difficult. As an effort to standardize medical terms, the Unified Medical Language Systems (UMLS) [3] maintains a very valuable resource of controlled vocabularies. It contains over 200 million medical terms (also known as "medical concepts"). Each medical term is identified by a unique id known as a Concept Unique Identifier (CUI). The UMLS also records relations between medical terms. As a result, mapping biomedical text data to the UMLS and mining UMLS associated datasets often yield rich knowledge for many biomedical applications [4-8].

In order to effectively query or use the UMLS, one of the fundamental tasks is to correctly map a biomedical term to a UMLS concept. Currently, there are a number of publicly available tools to achieve this goal. One notable approach is to use the official UMLS UTS service (UMLS Metathesaurus Browser) available on the UMLS official website (https://uts.nlm.nih.gov). Users are able to input a medical term and the system will return a query result. MetaMap [1], which has been developed and maintained by US National Library of Medicine, has become a standard tool in mapping biomedical text to the UMLS Metathesaurus. cTAKES [2] is an open- source natural language processing system that can process clinical notes and identify named entities from various dictionaries, including the UMLS.

However, after having been using these tools in our research, we found that they do not work well in mapping medical terms that are just slightly different from the terms in the UMLS. For example, the UMLS Metathesaurus Browser, MetaMap, and cTAKES fail to process the query term "1-undecene-1-O-beta 2',3',4',6'-tetraacetyl glucopyranoside" even if it has only one character different (missing "-" between "beta" and "2") from the official UMLS concept "1-undecene-1-O-beta-2',3',4',6'- tetraacetyl glucopyranoside". This drawback makes it hard to handle many real world data such as Electronic Health Records, which contain a lot of noisy information including missing and incorrect data [9]. In addition, they often fail to handle long medical terms even if those terms are identical to the terms in the UMLS. For example, the Metathesaurus Browser cannot handle query terms with more than 75 characters, and sometimes cannot even accurately answer a query term that exactly matches a concept name in the UMLS (see discussions in the result section). MetaMap and cTAKES, on the other hand, often breaks down a long medical term into several shorter terms. For example, if we query MetaMap with a clinical drug "POMEGRANATE FRUIT EXTRACT 150 MG Oral Capsule", we get several UMLS concepts such as "C1509685 POMEGRANATE FRUIT EXTRACT", "C2346927 Mg++", and "C0442027 Oral", instead of this drug concept which has a unique CUI C3267394 in the UMLS. The situation becomes even worse when medical terms contain special characters, i.e., characters other than numbers or letters, such as "{", "}", " (",")","-", etc. For example, MetaMap completely fails to find any relevant CUI to the medical concept "cyclo(Glu(OBz)-Sar-Gly-(N-cyclohexyl)Gly)2". These drawbacks are very undesirable when handling biomedical texts. By studying the UMLS Metathesaurus, we found that a significant number of medical terms are quite long. About 10.7% of UMLS concepts contain at least 75 characters (including white spaces), and about 50.9% of UMLS concepts contains at least 32 characters. In addition, a large amount of medical terms contain special characters. More than 61.3% of UMLS concepts contain at least one special characters and about 11% of UMLS concepts contains at least 5 special characters. In fact, we found many special characters are optional in a medical term. For example, term "Cyclic AMP- Responsive DNA-Binding Protein" and term "Cyclic AMP Responsive DNA Binding Protein" both refer to the same concept "C0056695" in the UMLS Metathesaurus, though the latter is missing two "-". The UMLS handles a medical term with different names by including multiple common names in the Metathesaurus. Given the fact that in many cases special characters are optional, it is practically impossible to let Metathesaurus contain all possible names. Considering a UMLS concept with 20 special characters, if each special character may be replaced by a white space, then there are approximately 1 million aliases for this concept alone, not to mention that more than 0.3% of UMLS concepts contain 20 special characters or more.

This problem is in fact related to the classical spelling correction problem in which a misspelled word will be corrected to the most closely matched word. The classic measurement of dissimilarity between two words based on several distance functions, such as edit distance [10], hamming distance [11], and longest common subsequence distance [12,13]. Thus the spelling correction is essentially finding a valid word with the minimum distance to the misspelled word. Quite a few dynamic programming algorithms have been proposed to solve this problem. Readers can find a survey of these algorithms in [14]. In recent years, spelling correction has evolved to perform query corrections. This correction is often a task of context sensitive spelling correction (CSSC), where corrections will be geared towards more meaningful or frequently searched words [15]. Thus, it is a good idea to use the query log to assist the correction [16].

Unlike many query applications, it is not sufficient to return a frequently searched medical term that best matches the query based on search history, not to mention that such history data is often not available. Accurately identifying a specific biomedical term, such as a drug name or a chemical compound, is demanded by many biomedical applications. Given this consideration, classical spelling correction techniques are more preferable than the CSSC for matching biomedical terms to UMLS concepts. However, we found that the classical dynamic programming algorithm is too slow for this task because of the huge volume of terms in the UMLS Metathesaurus. In addition, it is unable to effectively handle a term with missing words (e.g., "gastro reflux" has a large distance to "gastro oesophageal reflux" though the two terms usually mean the same thing), or words not in their usual order (e.g., "lymphocytic leukemia chronic" has a large distance to "leukemia chronic lymphocytic").

The background described above motivated us to find an efficient and accurate medical term mapping method for the UMLS. To tackle this challenge, in this work we propose a Layered Dynamic Programming Mapping (LDPMap) approach to query the UMLS Metathesaurus.

## Methods

We use Longest Common Subsequence (LCS) to measure the similarity between two words. Given two words *A *and *B*, their similarity is defined as:

$$WordSimilarity\left(A,B\right)=2*\left|LCS\left(A,B\right)\right|/\left(\left|A|+|B\right|\right);$$

This similarity measure is a variation of the longest common subsequence distance [12]. We can observe that *WordSimilarity*(*A, B*) ranges between 0 and 1. In addition, *WordSimilarity*(*A, B*) =1 if and only if *A *and *B *are identical, and *WordSimilarity*(*A, B*) = 0 if and only if *A *and *B *share no common letters.

The function *WordSimilarity*(*A, B*) is the basic building block for LDPMap. In the UMLS, each concept is a sequence of words. We define the similarity between two concepts *α_n _*= (*A*_1_, *A*_2_, ..., *A*_n_) and *β*_m _= (*B*_1_, *B*_2_, ..., *B*_m_) as:

$$ConceptSimilarity\left(\alpha_{n},\beta_{m}\right)=max\left(\underset{\left(i,j\right)\in R}{\sum }WordSimilarity\left(A_{i},B_{j}\right)\right);$$

Similar to word similarity, in our query we will normalize the concept similarity by the number of words contained in each concept. We can observe that normalized concept similarity score ranges between 0 and 1. If two concepts are identical then this score is 1.

$$NormConceptSimilarity\left(\alpha_{n},\beta_{m}\right)=2*ConceptSimilarity\left(\alpha_{n},\beta_{m}\right)/(|\alpha_{n}|+|\beta_{m}|\text{)};$$

The key issue in the above definition is *R*, which is a matching relation between words in α_n_ and β_m_. We have two constraints on *R*, which leads to two different foci. *Constraint 1*: There do not exists two matching pairs (*i,j*), (*x,y*) in *R *such that *i*=*x *or *j*=*y*.

*Constraint 2*: In addition to constraint 1, for any two matching pairs (*i,j*), (*x,y*) in *R*, either *i*<*x *&&*j*<*y*, or *x*<*i *&&*y*<*j*.

**Figure 1:**
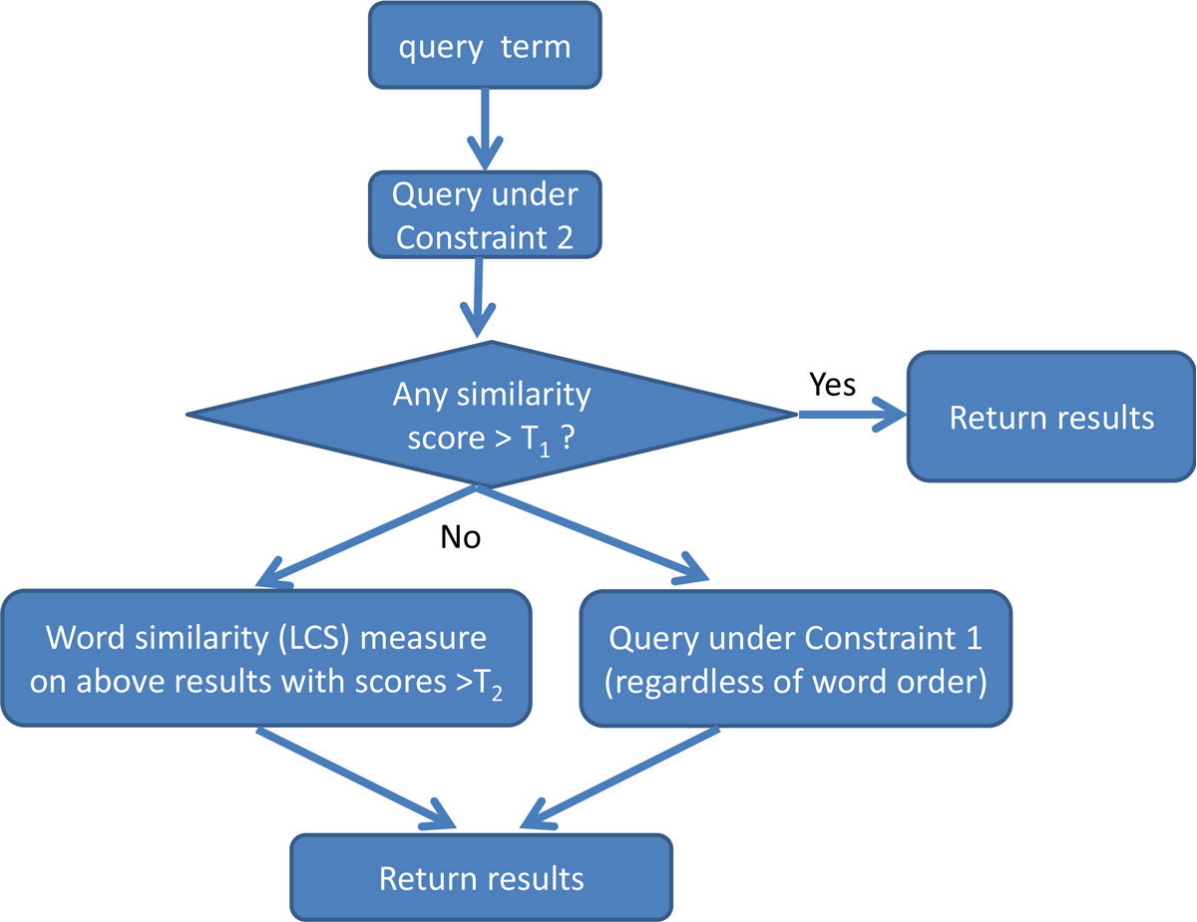
**A Comprehensive Query Workflow Using LDPMap**.

Constraint 1 converts the concept similarity problem into a maximum weighted bipartite matching problem [17]. Considering a bipartite graph built on two vertex sets *α_n _*and *β_m _*with word similarities being the edge weights, finding a highest score for concept similarity under Constraint 1 is equivalent to finding a maximum weighted matching for the bipartite graph. This model is particularly helpful for identifying the similarity between two terms regardless of their word ordering. We used this as one of the measurements in our final query workflow (Figure 1) and implemented this by maximal weighted matching.

In the following section, we will focus on concept similarity calculation under constraint 2, which regulates that the similarity comparison between two terms shall follow the word orders in those terms, similar to the LCS problem in which matching between two words shall follow the character orders. Thus, the concept similarity calculation problem can be considered as a macro level similarity calculation where each unit is a word instead of a letter as in the case of word similarity calculation. This model has a lot of advantages as we will see in the following section.

### Suboptimal structure of the concept similarity under constraint 2

Our next question is how to perform the concept similarity calculation. Unlike word similarity calculation in which each match outcome is a binary result (i.e., the same letter or a different letter), each match in the concept similarity calculation is a word similarity value between 0 and 1. The algorithm for the word similarity calculation cannot be applied to the concept similarity calculation. However, we find the concept similarity calculation also has a suboptimal structure as follows:

if *i*=0 or *j*=0

ConceptSimilarity(α_i_, β_j_) = 0

else

*ConceptSimilarity(α_i_, β_j_) = max(ConceptSimilarity(α_i-1_, β_j-1_) + WordSimilarity(A_i_, B_j_), ConceptSimilarity(α_i_, β_j-1_), ConceptSimilarity(α_i-1_, β_j_))*;

The above suboptimal structure is true because for any two words *A_i _* ε *α_i_, B_j _*∊ *β_j_*, there are at most three possible cases:

(1) (*i, j*) ∊ *R*, i.e, Both *A_i _*and *B_j _*are used in the matching. Then *ConceptSimilarity*(*α_i_, β_j_*) = *ConceptSimilarity*(*α_i-1_, β_j-1_*) + *WordSimilarity*(*A_i_, B_j_*);

(2) *B_j _*is not used in the matching, then *ConceptSimilarity*(*α_i_, β_j_*) = *ConceptSimilarity*(*α_i_, β_j-1_*);

(3) *A_i _*is not used in the matching, then *ConceptSimilarity*(*α_i_, β_j_*) = *ConceptSimilarity*(*α_i-1_, β_j_*).

Note that we do not consider it a valid case that neither *A_i _*nor *B_j _*is used in the matching. In this case, we can always choose to make them matching without violating Constraint 1 and result in a higher or at least equal concept similarity score.

## Main algorithms

Given the suboptimal substructure, we can design a dynamic programming algorithm to calculate the concept similarity score between two terms, on top of the LCS dynamic programming algorithm for calculating word similarity. The two layers of dynamic programming not only result in a method less affected by missing words or words in different orders, but also significantly increase the query speed as we will see below. These enable our searching method practically applicable to many biomedical applications.

The UMLS Metathesaurus (version used in this work: 2012AB) contains around 11 million records in its MRCONSO.RRF files. Each record is a medical term. For query purposes, we discard duplicate terms and non-English terms and result in about 6.87 million records. A term is considered duplicate if both its CUI and name are identical to another term. However, among these 6.87 million records, there are only 1,874,573 unique words (white space is the delimiter). Thus concept similarity on a word basis saves a huge amount of redundant calculation otherwise needed by classic methods on a character basis. Correspondingly, in our method, we first pre-process the UMLS Metathesaurus into a word vector of unique words, and convert each UMLS concept, which consists of a list of words, into a list of indices with regard to the word vector. Procedure LDPMap-Preprocessing is the pseudo code.

Procedure LDPMap-Preprocessing ( )

1: **for ***i*=1: length (*Metathesaurus*)

2:   *Word_Vector = Word_Vector *∪ *Metathesaurus *[*i*];

3: **endfor**

4: **for ***i*=1: length (*Metathesaurus*)

5: **for **j = 1: length (*Metathesaurus*[*i*])

    *WordIndex_vector *[*i, j*] = the index of *Metathesaurus*[*i, j*] in *Word_Vector;*

6: **endfor**

7: **endfor**

8: **return ***Word_Vector, WordIndex_vector*;

We process a query using the Algorithm LDPMap_Query. When a query process starts, we first build a word similarity matrix between the query term and the word vector (Line 1-5), using the *WordSimilarity *function defined above. Then we build a concept score vector between the query term and 6.87 million UMLS Metathesaurus concepts (Line 6-8). The construction of the concept score vector uses the *WordSimilarityMatrix *built previously so that there are no more word similarity calculations. In addition, it adopts a dynamic programming approach in Function *ConceptSimilarityScore*, owing to the suboptimal structure of the *ConceptSimilarity *function.

Algorithm LDPMap_Query (*query_term*)

1: **for ***i*=1: length (*query_term*)

2:   **for ***j*=1: length (*Word_Vector*)

3:     *WordSimilarityMatrix*[*i, j*] = *WordSimilarity*(*query_term*[*i*], *Word_Vector*[*j*]);

4:   **endfor**

5: **endfor**

6: **for ***i*=1:length(*Metathesaurus*)

7:   *ConceptScore_Vector*[*i*] = *ConceptSimilarityScore*(*WordIndex_vector*[*i*]);

8: **endfor**

9: **return **Concepts in Metathesaurus corresponding to top scores in *ConceptScore_Vector*;

Function ConceptSimilarityScore ( *WordIndex*)

1: **for ***i*=2:*x*+1

2:   **for ***j*=2:*y*+1

3:     *S*(*i, j*) = *WordSimilarityMatrix*[*i*-1, *WordIndex*[*j*-1]];

4:     **if ***S*(*i, j*)+*S*(*i*-1, *j*-1) >*max *(*S*(*i*-1, *j*), *S*(*i, j*-1));

5:       *S*(*i, j*)= *S*(*i, j*)+*S*(*i*-1, *j*-1);

6:     **else if ***S*(*i*-1, *j*) >*S*(*i, j*-1)

7:       *S*(*i, j*)=*S*(*i*-1, *j*);

8:     **else**

9:       *S*(*i, j*)=*S*(*i, j*-1);

10:     **endif**

11:   **endfor**

12: **endfor**

13: **return **2*S(*x*+1, *y*+1) / (*x*+*y*) ;

## A running example

To facilitate the understanding of our method, we provide a simple running example of our method in Tables 1 and 2. Assume the input query term is "gastro reflux". The Algorithm LDPMap_Query will first build a WordSimilarityMatrix between this query term and the word vector of Metathesaurus. Results were partially shown in Table 1.

**Table 1:** An example of *WordSimilarityMatrix *constructed for query term "gastro reflux".

		*Word Vector *of Metathesaurus						
	
		...	gastro (at i)	...	Oesophageal (at j)	...	reflux (at k)	...
**Query term**	gastro	...	1 (gastro)	...	0.235294 (so/ga)	...	0.166667(r)	...
	
	reflux	...	0.166667 (r)	...	0.235294 (el)	...	1(reflux)	...

After the WordSimilarityMatrix is available, the Algorithm LDPMap_Query will calculate the concept similarity scores between the query term and UMLS concepts by dynamic programming. The calculation will refer to WordSimilarityMatrix for word similarity score instead of calculating it again. An example of a concept similarity calculation is given in Table 2.

**Table 2:** An example of calculating the concept similarity score between the query term "gastro reflux" and the UMLS concept "gastro oesophageal reflux" for the *ConceptScore_Vector *construction.

		UMLS concept	gastro	oesophageal	reflux
		
		word index	i	k	j
**query term**	order		0	0	0

gastro	1	0	1	1	1

reflux	2	0	1	1.23594	2

The calculation will refer to the *WordSimilarityMatrix *as shown in Table 1. The normalized final similarity score is 2*2/(2+3)=0.8.

## Complexity analysis

The LDPMap method is much faster than the classic LCS-based word similarity calculation by treating the query term and each UMLS concept as one single word, as demonstrated in our empirical study. The classic LCS-based word similarity calculation uses dynamic programming on a character basis while we use two layers of dynamic programming, one on a character basis and the other on a word basis. To understand the analytical reason behind this speedup, let us make some simple assumptions. Assume the UMLS Metathesaurus contains *M *unique concepts, and each concept or query term contains *t *words, and each word has *d *characters. Also assume UMLS Metathesaurus contains *K *unique words. Then, the classic LCS-based word similarity calculation takes approximately O(*t^2^d^2^M*) time to handle a query. However, LDPMap method takes approximately O(*td^2^K*+*t^2^M*) time to handle this query. It is easy to observe that *K*<<*tM*. This explains why LDPMap is much more efficient. In the following, we will see that our LDPMap approach can be further sped up with the pipeline technique.

## Speeding up LDPMap with the pipeline technique

In building the *WordSimilarityMatrix *and *ConceptScore_Vector*, the dynamic programming method has been used for around 1.87 million times and 6.87 million times, respectively. It is interesting to find out if there are repeated calculations that can be reused to speed up the LDPMap method. By studying both the word vector and the Metathesaurus, we found the former has a lot of repeated prefixes among words (e.g. words "4-Aminophenol", "4-Aminophenyl"), and the latter has a lot of repeated prefix words among concepts (e.g. C1931062 ectomycorrhizal fungal sp. AR-Ny3, C1931063 ectomycorrhizal fungal sp. AR-Ny2). Thus, by lexicographically sorting the word vector and the Metathesaurus, we can use this information to save a lot of calculation in the LDPMap approach as follows:

(1) In calculating *WordSimilarityMatrix*, Given a word *A*, if it has *p *common prefix letters with the previous word *B*, the dynamic programming only needs to start from *p*+1 iteration because the previous *p*+1 columns of the dynamic programming table are exactly the same as the previous results.

(2) In calculating *ConceptSimilarityScore*, Given a concept *α*, if it has *q *common prefix words with the previous concept *β*, the dynamic programming only needs to start from *q*+1 iteration because the previous *q*+1 columns of the dynamic programming table are exactly the same as the previous results. That means, the for loop in Line 2 of Function *ConceptSimilarityScore *shall start with *j*=*q*+2.

The mechanism of the speedup technique can be described as a pipeline technique because a computation result can be passed down and partially reused by the subsequent computation. In the empirical study, we will see that the pipeline technique significantly improves the LDPMap speed.

## A comprehensive query workflow using LDPMap approach

Given the above solutions to the concept similarity problem under Constraints 1 and 2, we will design a comprehensive query workflow for mapping a query term to UMLS concepts. Our query workflow needs to consider multiple types of input variations and errors. Other than missing words and words in different orders that can be properly handled by concept similarity problem formulation, we need to consider another situation when two words are merged together. In this situation, the concept similarity modelling does not fit well because it is on a word basis. Therefore it is preferable to use the classic LCS method. However, as we pointed out above, the classic LCS method is too slow for the UMLS Metathesaurus. Fortunately, we found that we can leverage concept similarity solutions, outputting a list of concepts with similarity score greater than a threshold. When we set the threshold to be 0.35, in most cases it is able to output concepts that are similar with the query term regardless of the word merging issues. The number of outputted concepts is much smaller than the size of UMLS Metathesaurus; thus applying the LCS method on this small subset is much faster than on the whole UMLS Metathesaurus. The query workflow is illustrated in Figure 1.

In the query workflow, we first calculate concept similarity scores under Constraint 2 between the query term and all UMLS concepts. If there are concepts with scores higher than threshold *T*_1_, we output the results and the query completes. Otherwise, we save any concepts with scores higher than threshold *T*_2_ as *SET*(*T*_2_), and then perform two additional queries: (1) calculate word similarity between the query term and each concept in *SET*(*T*_2_) by treating the query term and each concept as one single word; (2) calculate the concept similarity scores under Constraint 1 between the query term and all UMLS concepts. Finally, we merge and output the results from (1) and (2). The number of results outputted is adjustable. An application can choose to output concepts with scores higher than a threshold, or only the top ranked concepts.

## Results

To understand the actual performance of LDPMap, we implemented it in C++, and subjected it to two sets of empirical studies. In summary, the results demonstrate that LDPMap method performs much better than available methods in terms of query speed and effectiveness. All experiments were carried out on Linux cluster nodes with 2.4GHz AMD Opteron processors. For the LDPMap query workflow, we set two parameters *T*_1 _= 0.8 and *T*_2 _= 0.35.

### Query speed comparison

**Figure 2:**
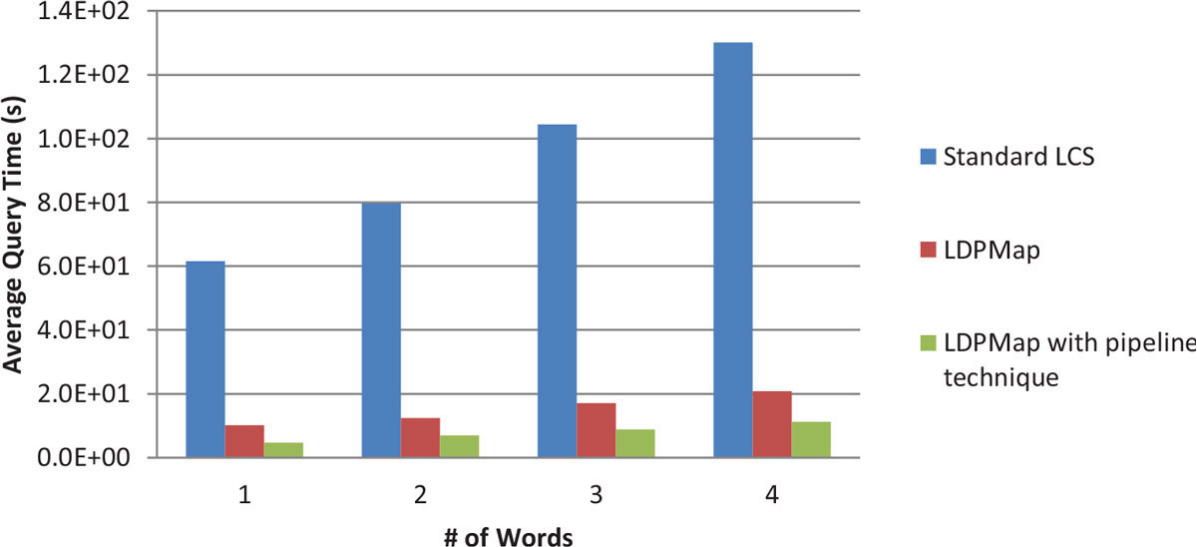
**Query time of LCS, LDPMap and LDPMap pipeline on randomly chosen 1000 medical concepts**.

We would like to know how fast LDPMap handles query in comparison with the standard LCS method which treats the query term and each UMLS concept as a single word, and how effective the pipeline technique for the LDPMap is. Therefore, we test the three algorithms, LCS standard, LDPMap (LDPMap_Query Algorithm) without the pipeline technique, and LDPMap algorithm with the pipeline technique, on four sets of medical concepts randomly chosen from the UMLS Metathesaurus. The first set consists of 1000 single-word medical concepts. The second, third and fourth sets consist of 1000 two-word, 1000 three-word, and 1000 four-word concepts, respectively. The results are shown in Figure 2.

From Figure 2 we can observe that the LDPMap algorithm is much faster than the standard LCS. In addition, the standard LCS method is susceptible to the word numbers in a query term while the LDPMap method is much more stable. This result is consistent with the above complexity analysis. In addition, the LDPMap with the pipeline technique significantly speeds up the basic LDPMap method. This confirms our intuition that the pipeline technique saves huge amounts of redundant computation thus improving the efficiency of the LDPMap method. As a result, we can see that in this set of experiments LDPMap with pipeline techniques on average answers a query in less than 1 second. However, the standard LCS method takes about 1 to 2 minutes in answering a query, which makes it virtually unacceptable for many biomedical applications, which can require near real-time responses, or when processing large amounts of data. In addition to the slow query time, the standard LCS is not good at processing query terms with missing words or words in different orders, as we have discussed above.

It is worthwhile to note that even for one word query, LDPMap method is significantly faster than LCS, though the concept similarity is exactly the same as the word similarity in this case. This is because the LDPMap pre-processed the UMLS terms on a word basis and built an efficient index. The similarity measurement is not directly on the UMLS terms but on words and the index which saves a lot of computational cost. In contrast, the LCS will handle the similarity measurement directly over every UMLS term. This can also be explained by our complexity analysis above. When *t*=1 (*t *is the number of words in a query), LCS complexity is O(*d^2^M*) while the LDPMap is O(*d^2^K*+*M*). Since *K*<<*M*, we conclude that LDPMap is much faster than LCS.

Next, we would like to know how effective LDPMap handles queries, especially when the query terms are slightly different than the terms in the UMLS Metathesaurus.

### Query effectiveness comparison

To understand how effective LDPMap (referring to LDPMap query workflow in this set of experiments) handles queries with name variations and errors, we used two available methods, UMLS Metathesaurus Browser and MetaMap as benchmarks. In a cursory examination of cTAKES, we found that it exhibited similar characteristics to MetaMap in its ability to handle name variations and errors and therefore we have excluded it from comparison. Since the study on UMLS Metathesaurus Browser requires manually inputting terms and checking the results, we have to limit the query test to manageable numbers. In addition, since the UMLS Metathesaurus Browser cannot accept a query term with more than 75 characters, we limit all query terms in our test to be no more than 75 characters. Given the above situations, and considering the fact that more than 50% of UMLS concepts contain at least 32 characters, we randomly chose 100 medical concepts with 32-75 characters from the UMLS Metathesaurus.

The 100 medical concepts are divided into two groups. The first group consists of 50 concepts with no special characters (i.e., characters other than letters and numbers), and the second group contains 50 concepts with 5 or more special characters. The two groups are for two different testing purposes.

*Group 1*: We will use group 1 to test how effective the query workflow handles pure English name terms, and English name terms with input errors, variations, and typos. Thus, in addition to querying the original names, we also query the names with 1, 2, 3, and 4 character variations. Character variations are generated randomly in this study, including (1) deleting a character, (2) replacing a character, (3) merging two words, i.e., deleting the white space between two words.

*Group 2*: We will use group 2 to test how effective the query algorithm is in handling many professional medical terms, which may contain a good number of special characters, such as chemical compounds and drugs. To simulate the name variations that frequently appear in these terms, we randomly apply 1, 2, 3, and 4 character variations, including (1) deleting a special character, (2) replacing a special character by a white space.

To complement the above test groups, we use the following group to test how effective the query algorithm handles short terms which may be queried commonly in real situation.

*Group 3*: We randomly picked 100 medical concepts with 5-31 characters. Since many of these concepts are quite short, we only apply 1 and 2 random character variations, including (1) deleting a character, (2) replacing a character, (3) merging two words.

In these experiments, we found that MetaMap often output multiple matching results but there are no ranks of these results. In contrast, the UMLS Metathesaurus Browser usually outputs a list of ranked concepts, and LDPMap can be configured to output the top *k *(*k*>=1) ranked concepts.

Thus, to be as fair as possible, we use two criteria to measure the correctness of a query:

*Criterion 1*: A query is correct if the original term appear (1) in top 25 ranked concepts (i.e., in the first page of the result) by the UMLS Metathesaurus Browser; (2) in the top 25 ranked concepts by LDPMap; (3) in the result of MetaMap.

*Criterion 2*: A query is correct if the original term appears (1) as the top ranked concept by UMLS Metathesaurus Browser; (2) as the top ranked concept by LDPMap.

Criterion 1 indicates if the query processing mechanism is able to handle the query with reasonable accuracy. Criterion 2 is much stringent and it indicates whether a method can be applied to applications require high accuracy.

**Figure 3:**
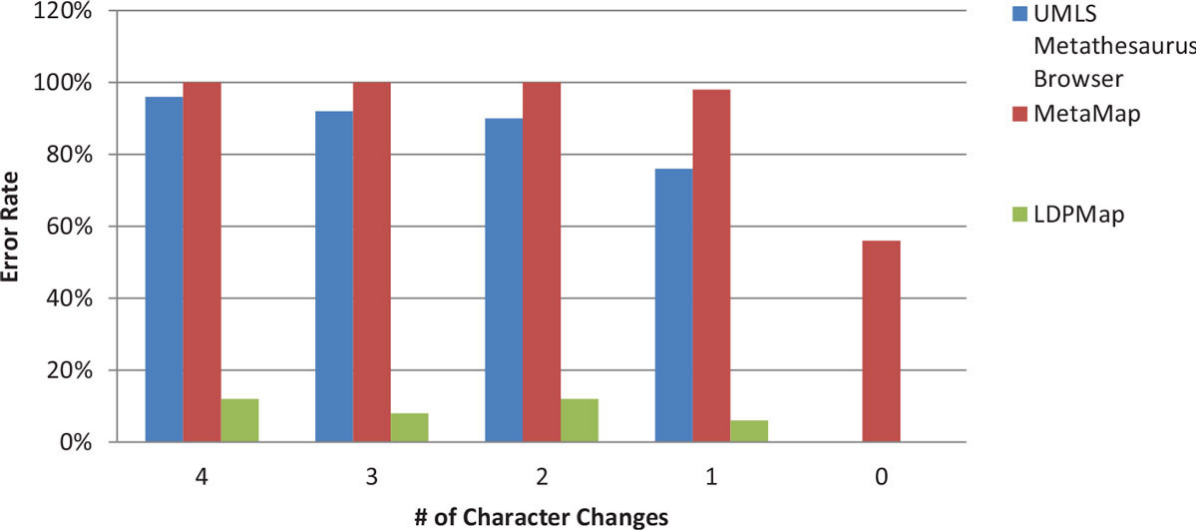
**Correctness comparison on LDPMap, UMLS Metathesaurus Browser, and MetaMap for Group 1 using Criterion 1**.

**Figure 4:**
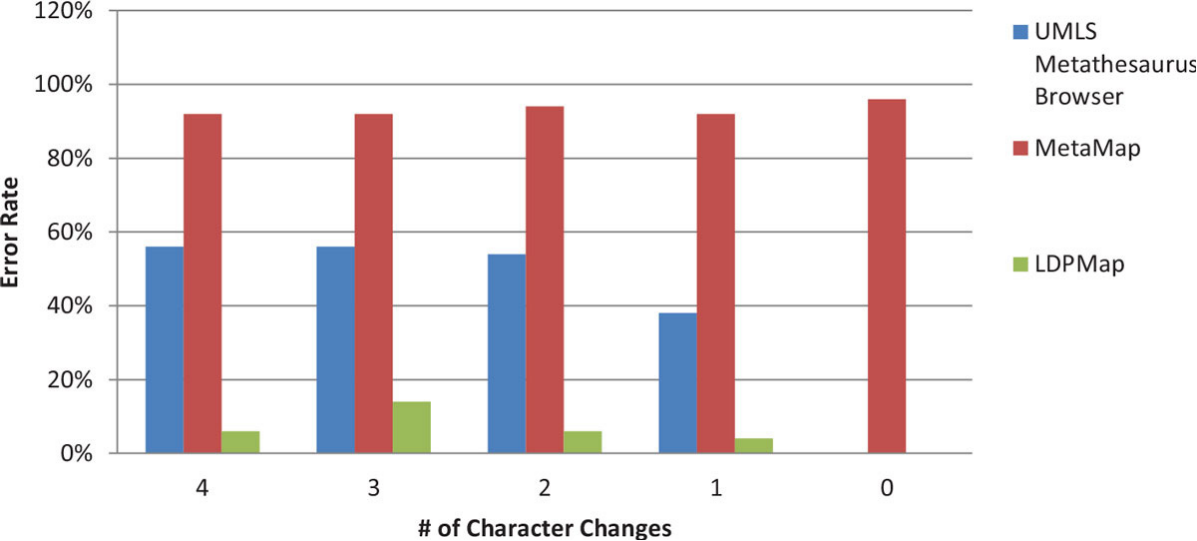
**Correctness comparison on LDPMap, UMLS Metathesaurus Browser, and MetaMap for Group 2 using Criterion 1**.

Figures 3 and Figure 4 are the error rate for the two groups of experiments, under Criterion 1. From both figures, we can clearly see that the LDPMap approach has very few errors among all tests. In comparison, the UMLS Metathesaurus Browser and MetaMap's error rate are quite high especially when multiple characters changes are present. MetaMap has a considerable error rate even when querying the original terms (0 characters changes). This may owe to the text processing mechanism of MetaMap. Since MetaMap is targeted at finding medical terms from a biomedical text, it leverages a combination of part-of-speech tagging, shallow parsing, and longest spanning match against terms from the SPECIALIST Lexicon before matching terms against concepts in the UMLS. Therefore, it tends to decompose longer spans of text and medical terms into several shorter medical terms.

**Figure 5:**
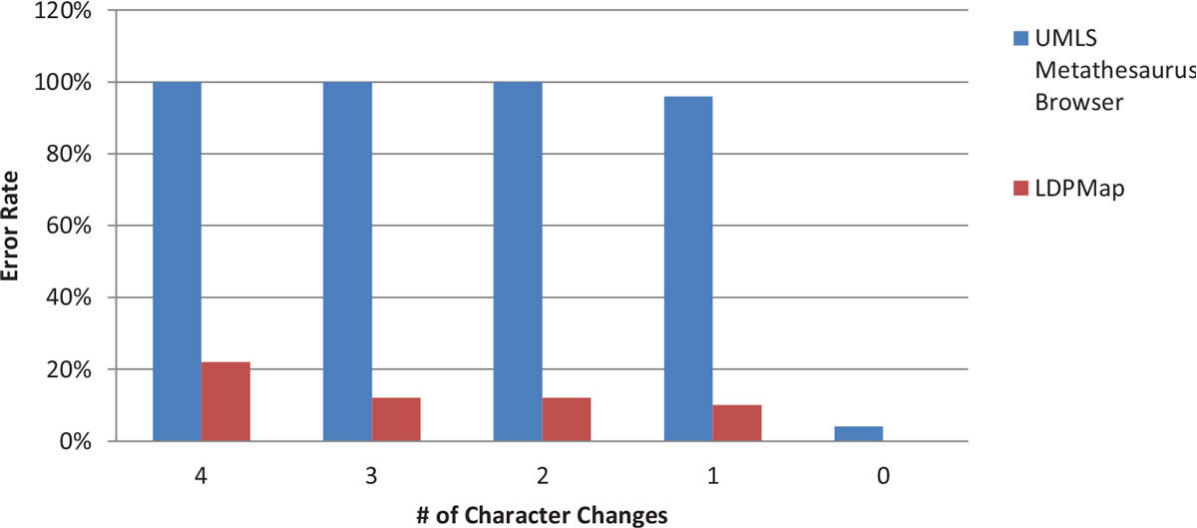
**Correctness comparison on LDPMap and UMLS Metathesaurus Browser for Group 1 using Criterion 2**.

**Figure 6:**
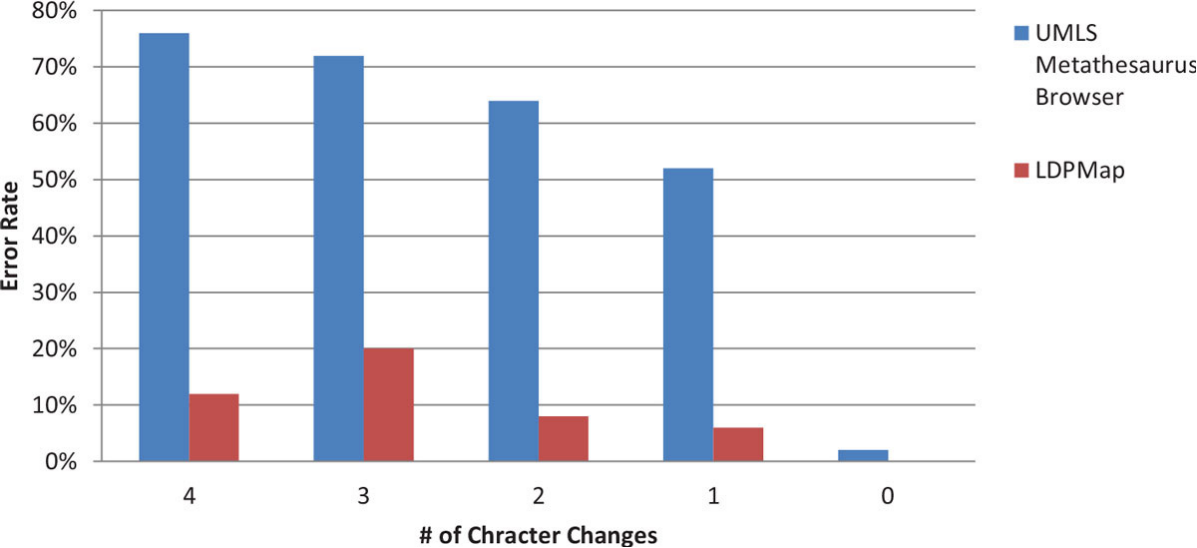
**Correctness comparison on LDPMap and UMLS Metathesaurus Browser for Group 2 using Criterion 2**.

Figure 5 and Figure 6 are the error rates for the two groups of experiments, under Criterion 2. Since MetaMap usually outputs multiple concepts without ranking, we exclude MetaMap from the Criterion 2 measurement. From these two figures, we can observe that the error rate of the UMLS Metathesaurus Browser is much higher in comparison with the measurement of Criterion 1. Quite surprisingly, there are some errors even when querying a few original terms (such as " Distal radioulnar joint"). This suggests that UMLS Metathesaurus Browser is not suitable for query processing for applications that have a high-accuracy demand. In contrast, the LDPMap still has a very low error rate, on average less than 5% across the 0-5 character changes, and free of errors in querying the original terms.

**Figure 7:**
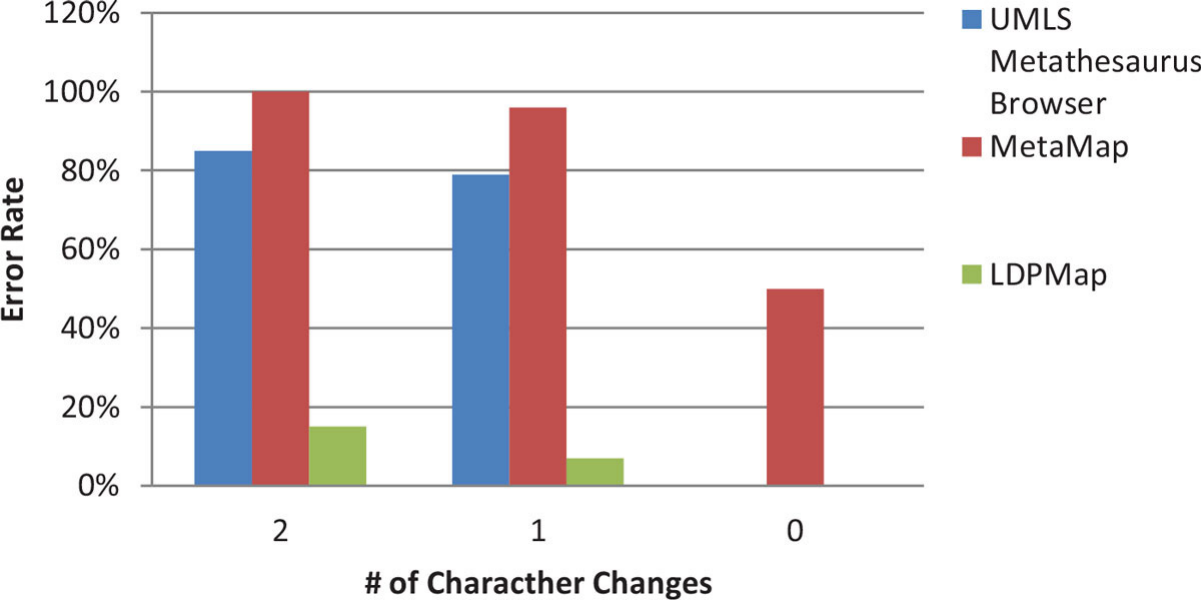
**Correctness comparison on LDPMap, UMLS Metathesaurus Browser, and MetaMap for Group 3 using Criterion 1**.

**Figure 8:**
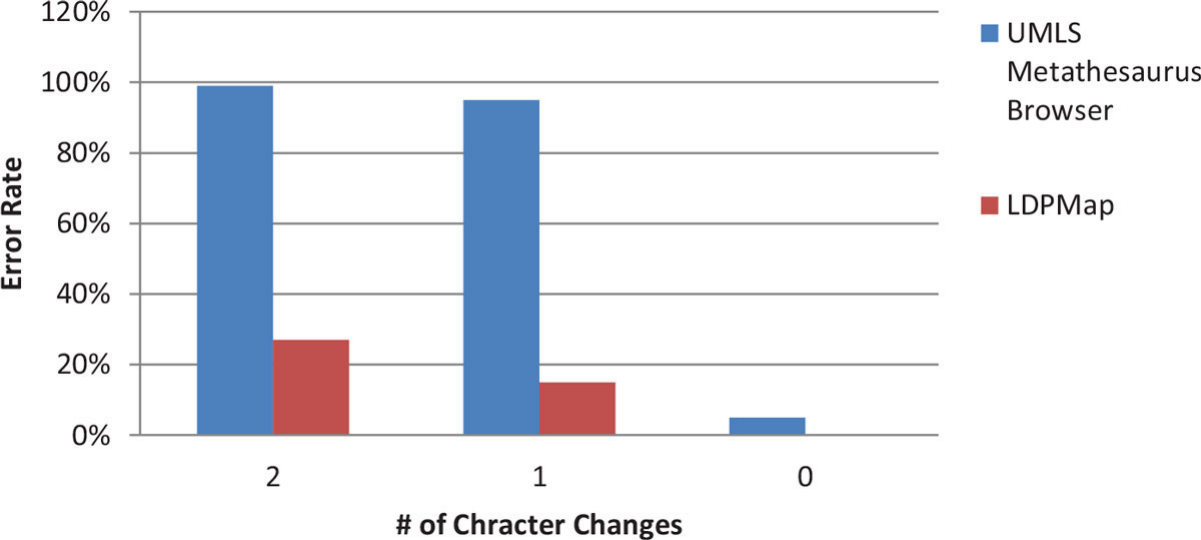
**Correctness comparison on LDPMap and UMLS Metathesaurus Browser for Group 3 using Criterion 2**.

From Figure 7 and Figure 8, we can see that the general performances of LDPMap, UMLS Metathesaurus Browser, and MetaMap on short query terms are similar to their performances on long query terms. LDPMap still has a clear advantage over UMLS Metathesaurus Browser, and MetaMap. However, we noticed that LDPMap error rate reaches 27% for 2 character changes under Criterion 2. This is understandable because generally short terms contain fewer words than long terms, and the concept similarity measurement is less favoured. However, the parameter *T*_1 _can be used as an adjustment of preference between the concept similarity measurement and the word similarity measurement. By increasing *T*_1 _from 0.8 to 0.85, we observed that this error rate reduces from 27% to 20%. This demonstrates that LDPMap is flexible in handling both long and short term queries.

To provide some details on the medical concepts we used in this set of experiments, and the character changes applied. We list a few of them in Table 3. From this table, we can see that it contains concepts of different lengths. The randomly generated character variations cover several common cases of text data inaccuracy, including, misspellings, merging of two words, and special character omissions. From Table 4 we can see that MetaMap cannot handle them properly. Instead, it finds some concepts related to individual words in the query term. The UMLS Metathesaurus Browser does not do any better on them. In contrast, LDPMap correctly answered all these queries except for "AlbunexIectable Product". Although "Injectable Product" is not correct, it is at least closer to the original term than those returned by the UMLS Metathesaurus Browser and MetaMap. By reviewing the LDPMap approach, we conclude that this error can be eliminated if we increase the threshold *T*_1 _to a value such that word similarity (LCS) is used to measure the two terms. To confirm this, we increase *T*_1 _from 0.8 to 0.85, and LDPMap successfully returns the original term. However, a high *T*_1 _implies that LDPMap gives more preference to LCS-based similarity measurement than to concept similarity measurement defined above. Consequently, LDPMap will be less productive in handling real-world queries that contain incomplete medical terms (i.e., medical terms with missing words). It is quite evident that there does not exist one set of *T*_1 _and *T*_2 _that fits all situations. As a result, we will fine tune these parameters to leverage LDPMap in our future applications.

**Table 3:** Original terms and their randomly generated character variations

CUI	Name	Randomly generated 4 character variations
C3267394	POMEGRANATE FRUIT EXTRACT 150 MG Oral Capsule	POMGRAATE FRUIT EXTRdCT 150 MG Oral Casule

C3228202	Albunex Injectable Product	AlbunexIectable Product

C0505183	Lateral branch of dorsal ramus of fifth thoracic spinal nerve	LateMa branch of dorsal ramus of ifth thoracic gpinal nerve

C1459293	Sinorhizobium americanus	Sinokhizrbimamericanus

C1541607	gp100/IL-7/ISA-51/MART-1	gp100 IL 7ISA-51/MART1

C1352046	danthron 1.5 MG/ML / Pantothenic Acid 2.5 MG/ML Oral Suspension	danthron 15 MGML Pantothenic Acid 25 MG/ML Oral Suspension

C0040372	Benzenesulfonamide, N-(((hexahydro-1H-azepin-1-yl)amino)carbonyl)-4-methyl-	Benzenesulfonamide, N-(( hexahydro1H- azepin-1-yl amino)carbonyl-4-methyl-

C2714409	1-undecene-1-O-beta-2',3',4',6'-tetraacetyl glucopyranoside	1-undecene1-O-beta2,3',4',6-tetraacetyl glucopyranoside

**Table 4:** Query results for Table 3.

CUI	UMLS Metathesaurus Browser (concept ranked 1st by approximate match)	MetaMap	LDPMap
C3267394	C0030054 Oxygen	C0016767 Fruit, C2346927 Mg++, and 4 others	correct

C3228202	C1514468 product	C1704444 Product (Multiplicative Product) [Quantitative Concept] C1514468 product [Entity]	C0086466 Injectable Product

C0505183	C0007965 Chediak-Higashi Syndrome	C1706131 Branch(Branch(group)), C2700383 Branch(Branch of plant), and 6 others	correct

C1459293	No result	No result	correct

C1541607	C1512807 Integrated Learning System	C0020898 IL (Illinois (geographic location)), C1522481 MART-1 (MART-1 Tumor Antigen), and 2 others	correct

C1352046	C0029383 Osmium	C1129294 danthron 25 MG, C0439526 /mL [Quantitative Concept], and 3 others	correct

C0040372	C0265215 Meckel-Gruber syndrome	C0053169 benzenesulfonamide, C0441922 N+ (N+ (tumor staging)), and two others	correct

C2714409	C0030011 Oxidation	C0470206 +1 [Q uantitative Concept] C1417683 BETA2 (NEUROD1 gene), and 7 others	correct

## Conclusions

In the work we proposed LDPMap, a layered dynamic programming approach to efficiently mapping inaccurate medical terms to UMLS concepts. As a main advantage of the LDPMap algorithm, it runs much faster than classical LCS method therefore makes it possible to efficiently handle UMLS term queries. When similarity is counted on a word basis, LDPMap algorithm may yield a more desirable result than LCS. In other cases (such as word merging), it is possible that LCS query results are more preferable. Thus, in the comprehensive query workflow of LDPMap, the LDPMap method is complemented by LCS and adjustable by parameter *T*_1_. Different from using LCS alone, the LDPMap query workflow only applies LCS (when needed) to a very limited number of candidate terms thus achieves a very fast query speed.

In query effectiveness comparison, we observed that LDPMap has a very high accuracy in processing queries over the UMLS Metathesaurus involving inaccurate terms. In contrast, the UMLS Metathesaurus Browser has a very limited ability in handling these queries, though it can handle queries of accurate terms fairly well. Throughout the study, we also observed that MetaMap, in general, is not suitable for mapping long medical terms to the UMLS concepts as it focuses on extracting short medical terms from the query text.

Although LDPMap is very efficient in handling UMLS term queries, it has two major limitations. First, it cannot handle synonyms and coreferences. Fortunately, UMLS Metathesaurus often list a concept preferred names and synonyms so that LDPMap can work effectively in most cases, though the list may still not be complete. Second, it is not able to perform syntax-level processing as MetaMap does, such as extracting medical terms from an article. Whether it is possible to extend the LDPMap approach to overcome the two limitations remains an open question. In the future we would like to investigate this question and plan to use LDPMap as an efficient pre-processing tool to map medical terms to the UMLS concepts, and use the results in our knowledge discovery applications.

## Competing interests

The authors declare that they have no competing interests.

## Authors' contributions

KR implemented the LDPMap algorithm, carried out the experiments, and edited the manuscript. AL, AM, RM, and KH analyzed comparable methods, participated in the design of the study, and revised the manuscripts. YX led the project including development of the idea, design of the algorithms, and writing of the manuscript.

## Declarations

Publication of this article was funded by the Department of Biomedical Informatics, The Ohio State University, USA.

This article has been published as part of *BMC Medical Genomics *Volume 7 Supplement 1, 2014: Selected articles from the 3rd Translational Bioinformatics Conference (TBC/ISCB-Asia 2013). The full contents of the supplement are available online at http://www.biomedcentral.com/bmcmedgenomics/supplements/7/S1.
